# Supplementary material for: Newcastle Disease Virus (NDV) Oncolytic Activity in Human Glioma Tumors Is Dependent on CDKN2A-Type I IFN Gene Cluster Codeletion
Source: Cells. 2020 Jun 5;9(6):1405. doi: 10.3390/cells9061405 (PMC7349162; doi:10.3390/cells9061405)
Supplement: Supplementary file 1 [file cells-09-01405-s001.pdf]

cells

MDPI

Newcastle Disease Virus (NDV) Oncolytic Activity in Human Glioma Tumors is Dependent on CDKN2A–Type I IFN Gene Cluster Codeletion

Noemi García-Romero [1,2,3,†], Irina Palacín-Aliana [4,5,†], Susana Esteban-Rubio [6], Rodrigo Madurga [1,5], Sergio Rius-Rocabert [6,7,8], Josefa Carrión-Navarro [1,2,3], Jesús Presa [9], Sara Cuadrado-Castano [10], Pilar Sánchez-Gómez [11], Adolfo García-Sastre [10,12,13], Estanislao Nistal-Villan [6,7,*] and Angel Ayuso-Sacido [1,2,3,*]

[1] Faculty of Experimental Sciences, Universidad Francisco de Vitoria, 28223 Madrid, Spain; noemigromero@gmail.com (N.G.-R.), peps86@gmail.com (J.C.-N.)
[2] Brain tumour laboratory, Fundación Vithas, Grupo Hospitales Vithas, 28043 Madrid, Spain; ayusosacido@gmail.com
[3] Formerly: Fundación de Investigación HM Hospitales, HM Hospitales, 28015 Madrid, Spain
[4] Atrys Health, 08025 Barcelona, Spain; ipalacin@atryshealth.com
[5] Fundación de Investigación HM Hospitales, HM Hospitales, 28015 Madrid, Spain; rmadurga@fundacionhm.com
[6] Facultad de Medicina, Instituto de Medicina Molecular Aplicada (IMMA), Universidad San Pablo-CEU, 28668 Madrid, Spain; susana.rubio91@gmail.com (S.E.-R.); ser.rius.ce@ceindo.ceu.es (S.R.-R.)
[7] Microbiology Section, Dpto. CC, Farmacéuticas y de la Salud, Facultad de Farmacia, Universidad San Pablo-CEU, 28668 Madrid, Spain
[8] CEMBIO (Centre for Metabolomics and Bioanalysis), Facultad de Farmacia, Universidad San Pablo-CEU, 28668 Madrid, Spain
[9] Independent researcher, 28003 Madrid, Spain; jesus_l_presa@yahoo.es
[10] Department of Microbiology, Icahn School of Medicine at Mount Sinai, New York, NY 10029, USA; Global Health and Emerging Pathogens Institute, Icahn School of Medicine at Mount Sinai, New York, NY 10029, USA; sara.cuadrado@mssm.edu (S.C.-C.); adolfo.garcia-sastre@mssm.edu (A.G.-S.)
[11] Neurooncology Unit, Instituto de Salud Carlos III-UFIEC, Madrid 28220, Spain; psanchez@isciii.es
[12] Department of Medicine, Division of Infectious Disease, Icahn School of Medicine at Mount Sinai, New York, NY 10029, USA
[13] The Tisch Cancer Institute, Icahn School of Medicine at Mount Sinai, New York, NY 10029, USA
* Correspondence:Correspondence: ayusosacido@gmail.com; (A.-S.A.); estanislao.nistalvillan@ceu.es (E.N.-V.); Tel.: +34-686-966-904 (A.A.-S.); +34-913-724-714 (E.N.-V.)
† These authors contributed equally to this work.

Received: 24 April 2020; Accepted: 31 May 2020; Published: 02 June 2020

Supplementary Material

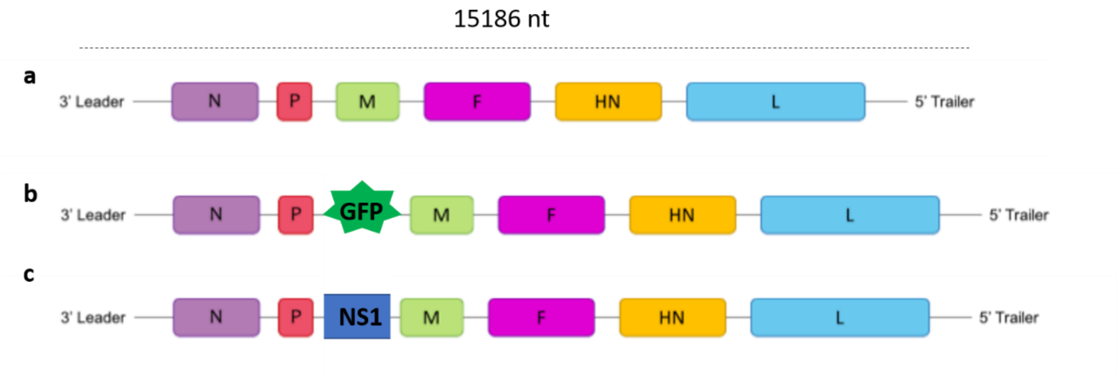

Figure S1. Schematic representation of rNDV viruses. **(a)** wt-NDV genome contains six genes that codifies for *nucleocapside* (*N*), *phospho* (*P*), *matrix* (*M*), *fusion* (*F*), *hemagglutinin-neuraminidase* (*HN*) and

large (l) proteins. **(b)** Green fluorescent protein (GFP) or **(c)** Influenza virus *NS1* gene were inserted between *P* and M genes.

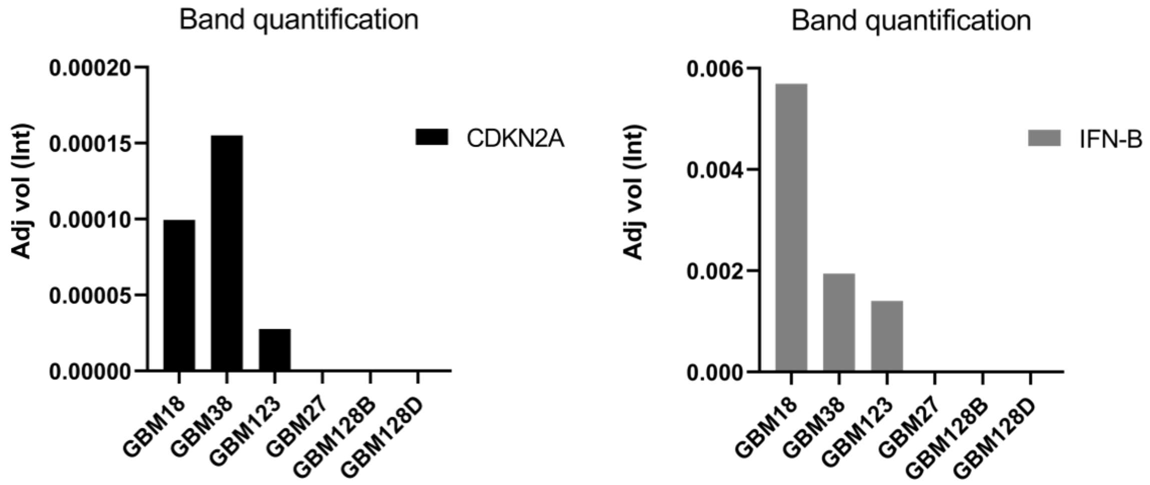

Figure S2. gDNA quantification of CDKNA and *IFN-β*. β-actin was used as reference control.

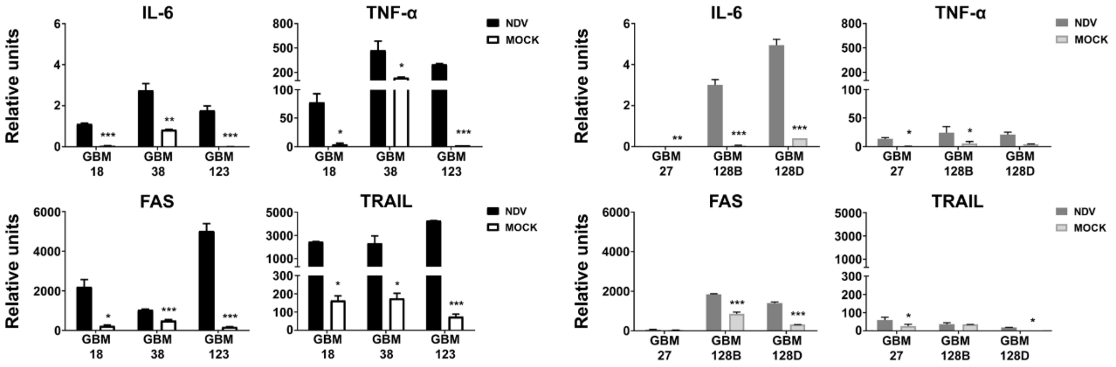

Figure S3. mRNA expression by qRT-PCR. CSCs were infected with NDV at high MOI (4), and RNA was extracted after 24 h. Data are the mean ± SD. * $p < 0.05$, ** $p < 0.01$, *** $p < 0.001$.

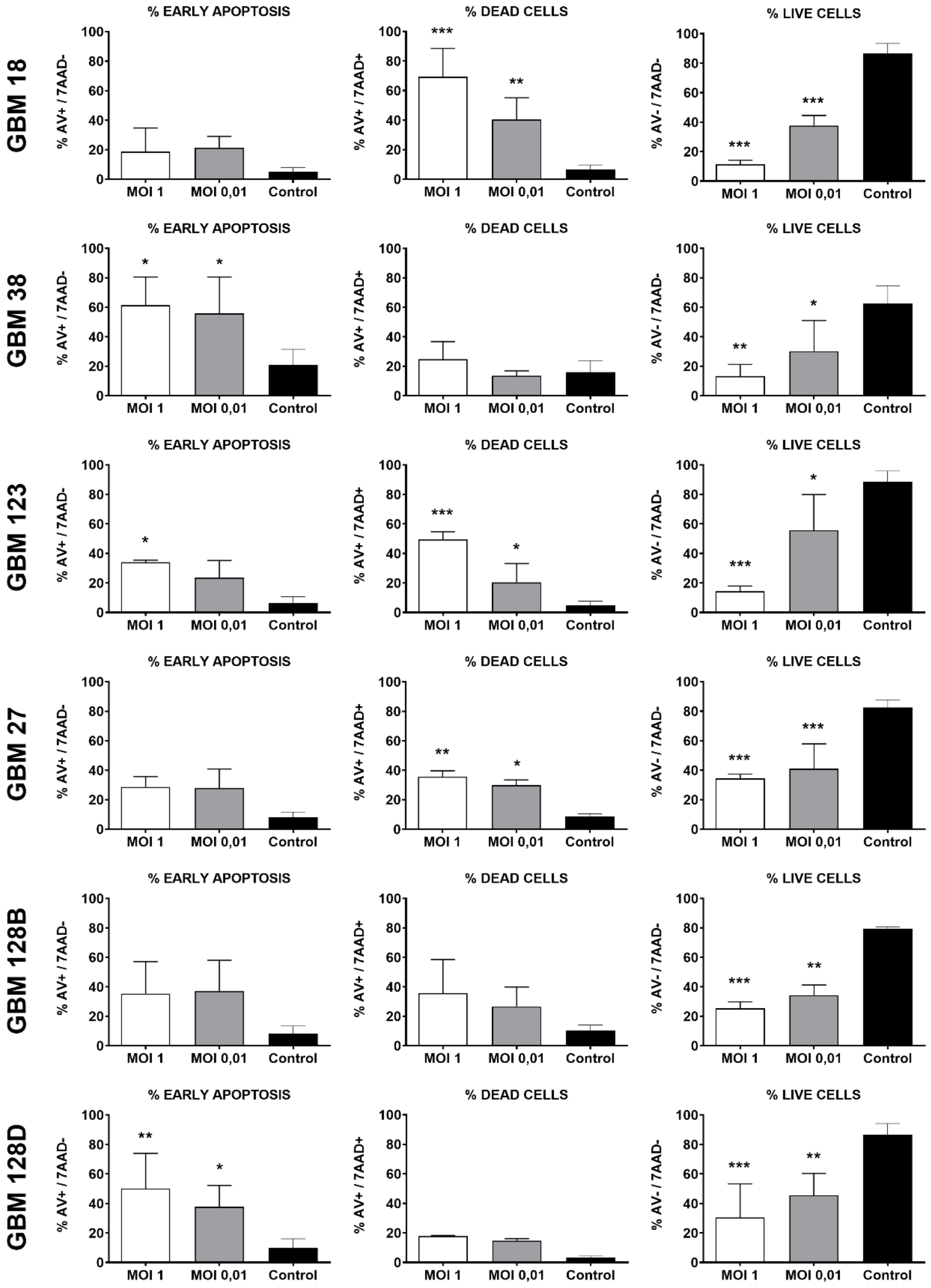

Figure S4. Annexin V/7AAD staining determined by Flow Cytometry at 120 h after NDV infection. Data are presented as the mean ± SD. * $p < 0.05$, **$p < 0.01$, *** $p < 0.001$.

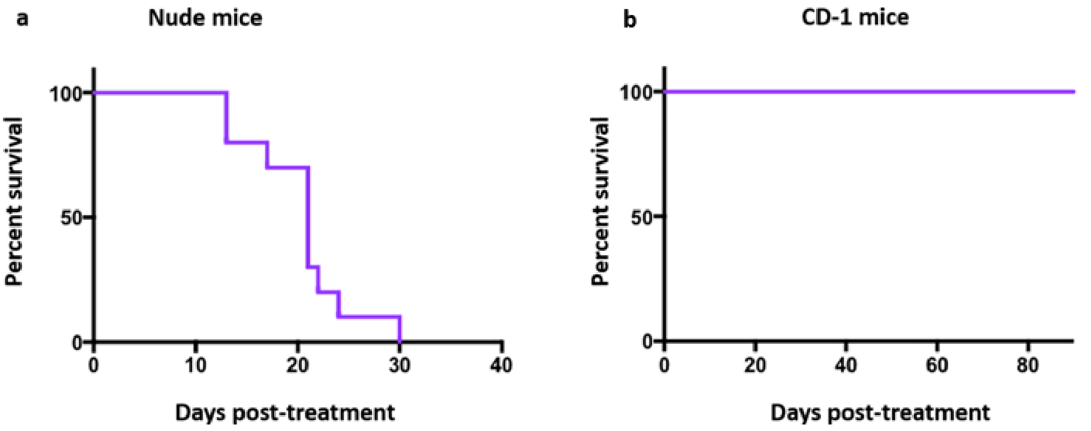

Figure S5. NDV toxicity assay. (**a**) 10 Nude and (**b**) 5 CD1 mice received a single intratecal dose of NDV. Animals were sacrified when their weight decreased more than 20% of their initial body weight.

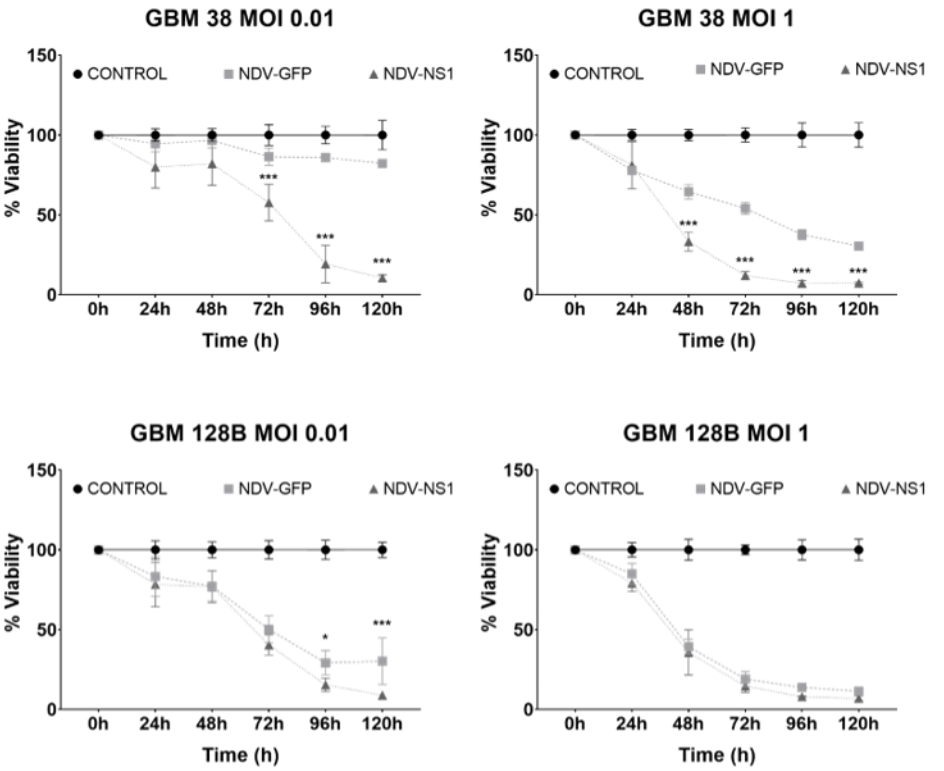

Figure S6. NDV-NS1 restored oncolytic activity in competent CSCs model. GBM38 and GBM128D were exposed to NDV-GFP and NDV-NS1 MOI 1 and 0.01. Cell viability was measured every 24 h during 120 h. Error bar represents Standard Deviation. * $p < 0.05$, *** $p < 0.001$.
